# Impact of Pulmonary Vein Isolation on Atrial Fibrillation Organisation: Correlation of Intracardiac and Surface Electrocardiogram Measures

**DOI:** 10.1111/jce.70044

**Published:** 2025-08-06

**Authors:** Nazifa Ahsan, Arunashis Sau, Joseph Barker, Rabiah Neerahoo, Norman Qureshi, Michael Koa‐Wing, Daniel Keene, Louisa Malcolme‐Lawes, David C. Lefroy, Nicholas W. F. Linton, Phang Boon Lim, Amanda Varnava, Zachary I. Whinnett, Prapa Kanagaratnam, Danilo P. Mandic, Nicholas S. Peters, Fu Siong Ng

**Affiliations:** ^1^ National Heart and Lung Institute, Imperial College London London UK; ^2^ Department of Cardiology Imperial College Healthcare NHS Trust London UK; ^3^ Department of Electrical and Electronic Engineering Imperial College London London UK

**Keywords:** ablation, AF organization, atrial fibrillation, entropy, pulmonary vein isolation, ventricular irregularity, ventricular variability

## Abstract

**Introduction:**

Electrical activity in atrial fibrillation (AF) ranges from organized focal drivers to multiple wavelet re‐entry. Understanding the effect of pulmonary vein isolation (PVI) on AF organization is clinically important, as it may optimize treatment strategies and outcomes. This study investigates the impact of PVI on AF organization and explores whether ventricular response regularity, measured from surface electrocardiograms (ECGs), reflects AF dynamics and electrophenotype.

**Methods:**

Patients undergoing first‐time PVI at Imperial College Healthcare NHS Trust between 2014 and 2022 were assessed pre‐ and post‐PVI. AF organization was quantified using Shannon entropy (ShEn) and Sample entropy (SampEn) from coronary sinus (CS) electrograms. Ventricular response regularity was evaluated using surface ECG RR interval (RRI) variability and SampEn.

**Results:**

PVI reduced ShEn and SampEn across all CS channels (e.g., SampEn CS 3‐4: pre‐ablation = 0.907 ± 0.512 vs. post‐ablation = 0.790 ± 0.446, *p* < 0.001). Atrial ShEn and SampEn were correlated with ventricular response both pre‐ and post‐ablation (e.g., correlations between atrial SampEn CS 3‐4 and the following ventricular metrics: percentage of RRIs > 50 ms difference: *r* = 0.077, *p* = 0.008; normalized mean RRI difference: *r* = 0.144, *p* < 0.001; and normalized SampEn: *r* = 0.168, *p* < 0.001).

**Conclusion:**

The reduction in atrial ShEn and SampEn post‐PVI indicates increased AF organization. The significant correlation between atrial entropy and ventricular variability suggests that AF organization affects ventricular response, assessed via surface ECG metrics. These findings highlight the potential of ECG‐based measures as proxies for intracardiac AF organization.

## 1 Introduction

Atrial fibrillation (AF) is characterized by uncoordinated atrial activation with irregularly irregular ventricular response [1]. Various mechanisms on how AF is sustained have been hypothesized. There are two overarching models of AF [2]. One model discusses how AF is driven by focal sources of rotational activity, which seem to localize to areas of fibrosis [2]. The contrary model discusses that AF is sustained by multiple electrical wavelets propagating randomly through atrial tissue, which is supported by studies showing an absence of localized sources [2, 3].

Another more contemporary explanation is the renewal theory in which AF is sustained by scroll waves, which undergo repetitive regeneration via the mechanisms of spiral wave breakup [2, 4, 5]. Wave breakup leads to the formation of secondary sources that propagate towards and merge with the original waves, resulting in new ones [6]. Ultimately, all of these mechanisms may be involved in sustaining AF where the AF electrophenotype exists on a spectrum determined by the underlying substrate. At the organized end, AF is driven by focal stable sources of rotational activity [2]. As this rotational activity becomes more dynamic with repetitive regeneration, AF becomes more disorganized, and at the most disorganized end, AF may be sustained by multiple wavelet re‐entry [2]. This spectrum of electrophenotype is likely to have implications for tailoring mechanism‐directed treatment strategies [2].

Pulmonary vein isolation (PVI) is the cornerstone of rhythm control for AF primarily because it reduces pulmonary vein ectopy [1, 7]. However, beyond this it is unclear how PVI affects AF organization dynamics from the remaining atrial triggers. It is logical that PVI resulting in a reduction in volume of electrically connected atrial tissue as well as underlying substrate modification might lead to changes in the action potential duration by altering the electrical load and conduction pathways preventing rapid re‐excitation and increasing the degree of organization of the AF electrophenotype [8, 9]. Indeed, PVI has been shown to influence atrial fibrillation cycle length which may contribute to improved reductions in AF burden and symptoms in the presence of recurrence following PVI [10, 11, 12, 13]. However, in highly fragmented atrial activity, cycle length may not reliably reflect underlying organization [10]. Furthermore, while measures such as temporal and spatial indices have demonstrated predictive value for AF termination and long‐term outcomes, conflicting findings highlight the need for better characterization of how PVI modifies AF electrophenotype [12].

In this study we aimed to investigate the impact of PVI on AF organization, with the hypothesis that AF becomes more organized following PVI using measures of entropy. We aimed to investigate the relationship between AF organization and ventricular response regularity to allow non‐invasive assessment and tracking of AF organization to facilitate rapid, non‐invasive AF electrophenotype assessment.

## 2 Methods

### 2.1 Study Population

In this retrospective cohort study, all patients undergoing first‐time catheter ablation for persistent and longstanding persistent AF in Imperial College Healthcare NHS Trust hospitals from February 2014 to January 2022 were considered for inclusion. AF was defined as persistent and longstanding persistent according to European current guidelines [1]. Only patients who were spontaneously in AF at the start of the procedure were included.

### 2.2 Ablation

PVI was performed in all patients by either radiofrequency or cryoablation. Adjunctive ablation procedures such as roof line ablation, posterior box isolation and complex fractionated atrial electrogram ablation were performed in a small proportion of patients as per electrophysiologist discretion.

### 2.3 Measurement of AF Organization

**Figure 1:**
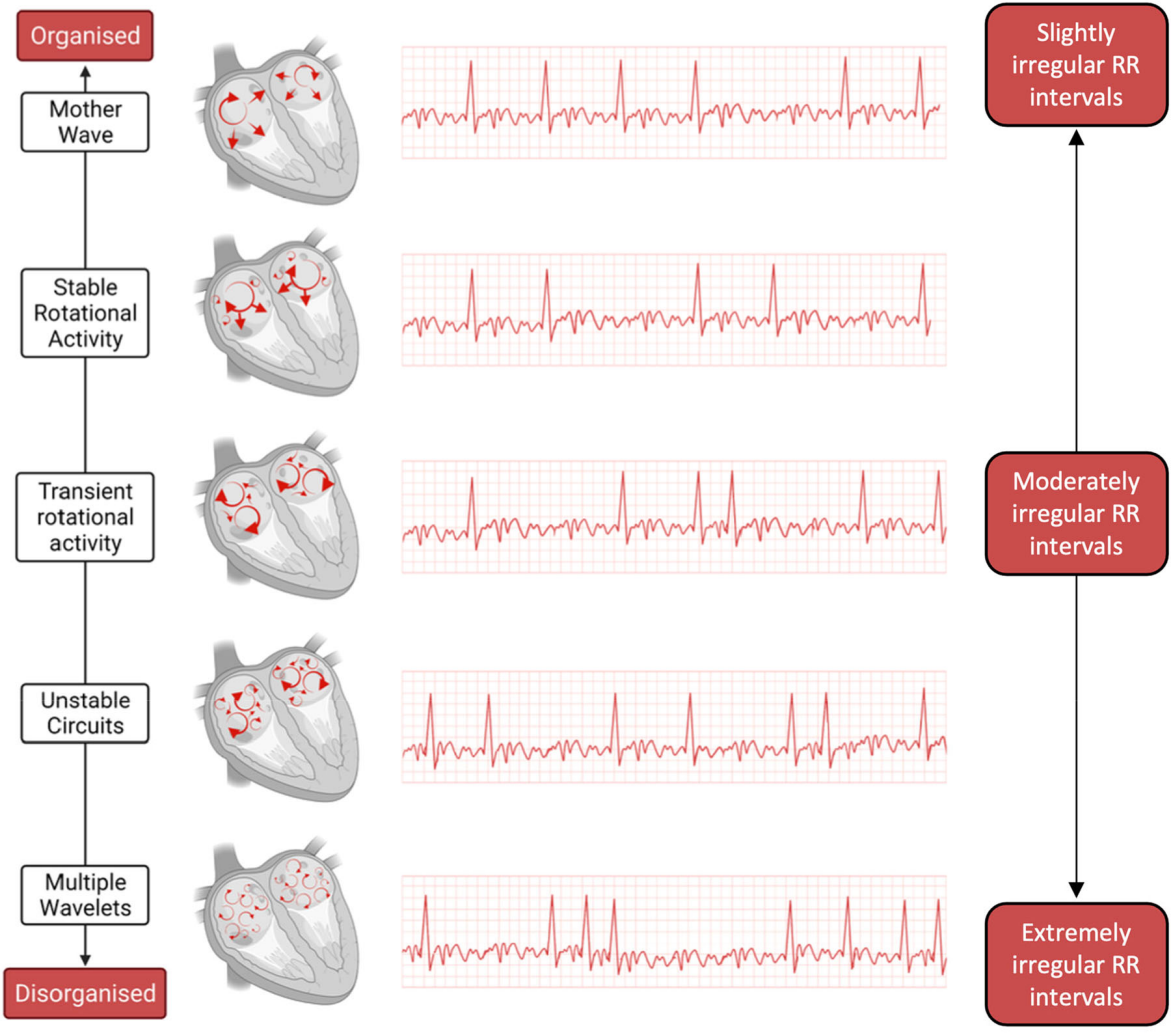
Schematic illustrating the spectrum of organization of atrial electrical activity and irregularity of RR intervals in atrial fibrillation (AF). At the organized end of AF, stable rotational activity in the atria leads to less irregular RR intervals compared to disorganized AF where multiple wavelets lead to chaotic atrial electrical activity and therefore extremely irregular RR intervals.

**Figure 2:**
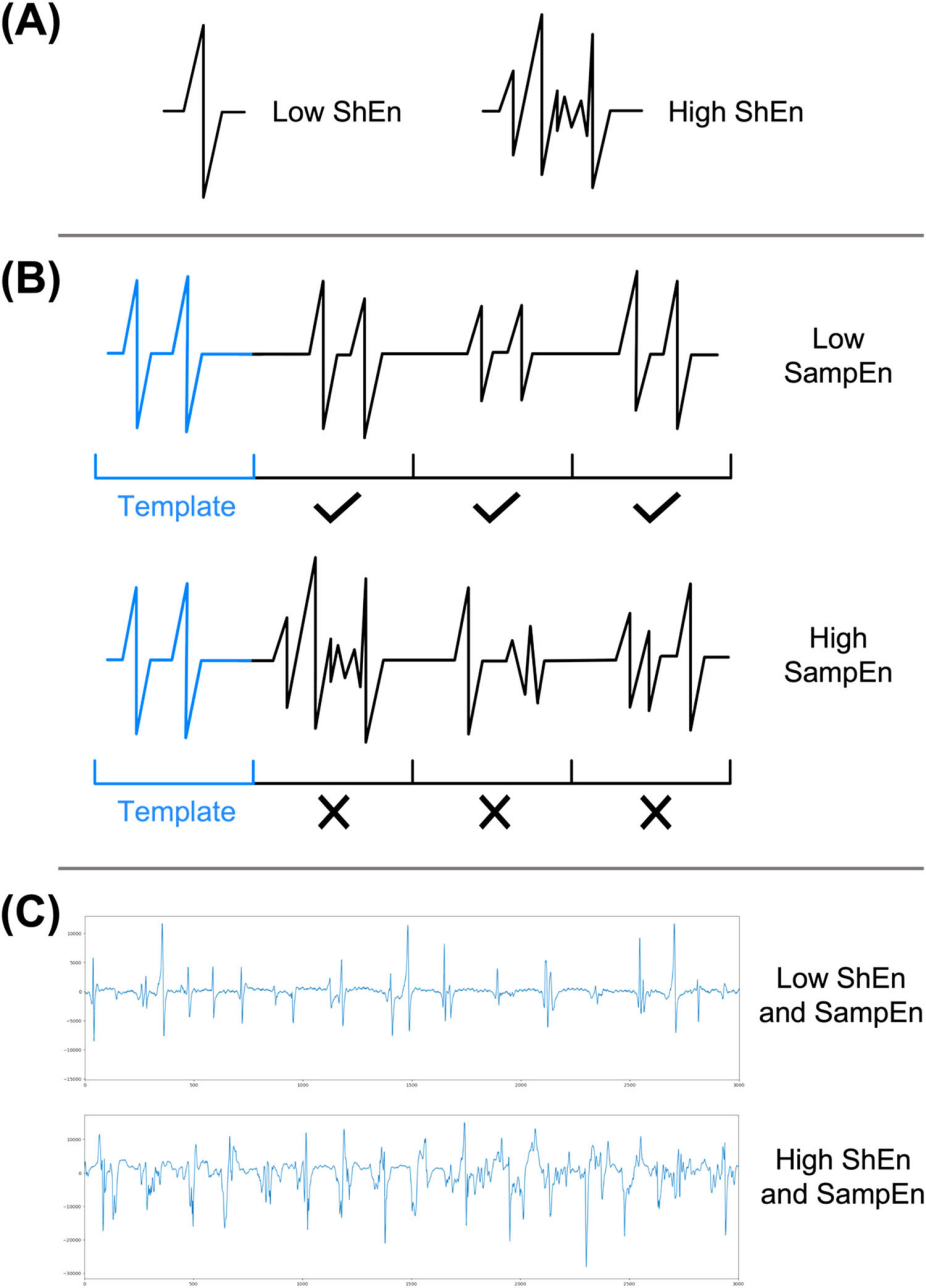
Schematic illustrating the concept of Shannon entropy (ShEn) and Sample entropy (SampEn). (A) A visual comparison of low and high ShEn. The electrogram on the left represents low ShEn since there is little amplitude variation, whereas the electrogram on the right represents high ShEn since there is a greater amplitude variation. (B) A visual comparison of low and high SampEn. The top electrogram trace illustrates low SampEn as the subsequent segments are similar to the template segment, resulting in a high number of matches (represented by the ticks). However, the bottom electrogram trace illustrates high SampEn as the subsequent segments are very different from the template segment, hence there are no matches (represented by the crosses). (C) Examples of coronary sinus (CS) electrograms of patients with atrial fibrillation. The top CS electrogram has regular signals, so it is an example of low ShEn and low SampEn, whereas the bottom CS electrogram has more complex and irregular signals, hence it is an example of high ShEn and high SampEn.

AF organization which exists on a spectrum (Figure 1) was measured using Shannon entropy (ShEn) and Sample entropy (SampEn) [14, 15]. ShEn was determined by the distribution of signal amplitudes of atrial electrograms, where more complex signals had a greater amplitude distribution, resulting in a higher ShEn (Figure 2A) [16, 17]. SampEn was determined by measuring the prevalence of similar patterns within the atrial electrogram, where a segment of the electrogram was taken as a template and compared to other segments [18]. Electrograms with irregular signals had a low number of matches, therefore a higher SampEn (Figure 2B) [18].

ShEn and SampEn were determined using atrial electrograms from up to 5 consecutive 50 s coronary sinus (CS) recordings taken before and after ablation. As a significant number of patients were cardioverted before completion of PVI, a compromise of ≥ 70% PVI completion was chosen. For patients who were cardioverted before ≥ 70% of the procedure was complete, entropy was measured pre‐ablation only. In the case of patients who were cardioverted before completion of the PVI but ≥ 70%, post‐ablation segments were exported from just before the point of cardioversion. SciPy version 1.8.0 was used for ShEn analysis, while the PyEEG version 0.0.2 was used for SampEn analysis [19, 20]. Separate ShEn and SampEn values were computed for each CS channel. Each ShEn value was determined from the whole 50 s of data. Each SampEn measurement was computed on 5 × 10 s period and averaged to give a SampEn value for the entire 50 s period. Atrial entropy values from the consecutive 50 s periods were averaged to give one pre‐ablation value and one post‐ablation value for each patient to compare atrial entropy before and after PVI. Atrial entropy values for each individual 50 s period were used to correlate with ventricular response regularity.

### 2.4 Measurement of Ventricular Response Regularity

Ventricular response regularity was evaluated using RRI variability and irregularity metrics derived from the surface ECG. RRI variability and irregularity were determined for the same 50 s observation periods within which atrial entropy was determined. The Hamilton algorithm was used for R wave detection, which was required to obtain the RRI measurements [21, 22].

RRI variability measures the range of the RRI values in the data set, whereas RRI irregularity evaluates the regularity of the patterns of the RRI values in the data series [23]. RRI variability was evaluated using the time‐domain measures: normalized mean RRI difference, standard deviation of RRIs (SD RRI), root mean square of successive RRI differences (rMSSD) and percentage of successive RRIs that differ from each other by more than 50 ms (pNN50); see *Supplement* for more details [24]. RRI irregularity was measured using SampEn. Normalized SD RRI and normalized SampEn were evaluated in addition to SD RRI and SampEn. Normalization was performed to account for the impact of heart rate on RRI variability and was performed by dividing the SD RRI or SampEn value by the mean RRI value [25].

### 2.5 Statistical Analysis

All variables are reported as mean ± standard deviation. Normality was evaluated using the D'Agostino‐Pearson test. Comparisons between pre‐ablation and post‐ablation atrial entropy were performed with paired *t*‐test. Correlations between atrial entropy and RRI variability were assessed using parametric Pearson correlation coefficient. Correlations between atrial entropy and RRI irregularity were evaluated using non‐parametric Spearman correlation coefficient. Corrections for multiple comparisons were not performed due to all comparisons being pre‐planned and to minimize type II errors, as previously described [26]. All statistical tests were two‐sided and all *p* values < 0.05 were considered statistically significant. Statistical analysis was performed using GraphPad Prism version 9.3.1 (San Diego, California, USA). This paper adheres to the Strengthening the Reporting of Observational Studies statement guidelines for reporting observational studies [27].

## 3 Results

### 3.1 Impact of PVI on Atrial Entropy

Atrial ShEn and atrial SampEn measurements were taken in the entire study population of 239 patients (mean age 62.8 ± 9.16, 72.8% male). Out of these 239 patients, both pre‐ and post‐ablation data were available for 171 patients (mean age 63.0 ± 8.96, 76.0% male). Comparisons between pre‐ and post‐ablation entropy values were carried out in these 171 patients.

**Figure 3:**
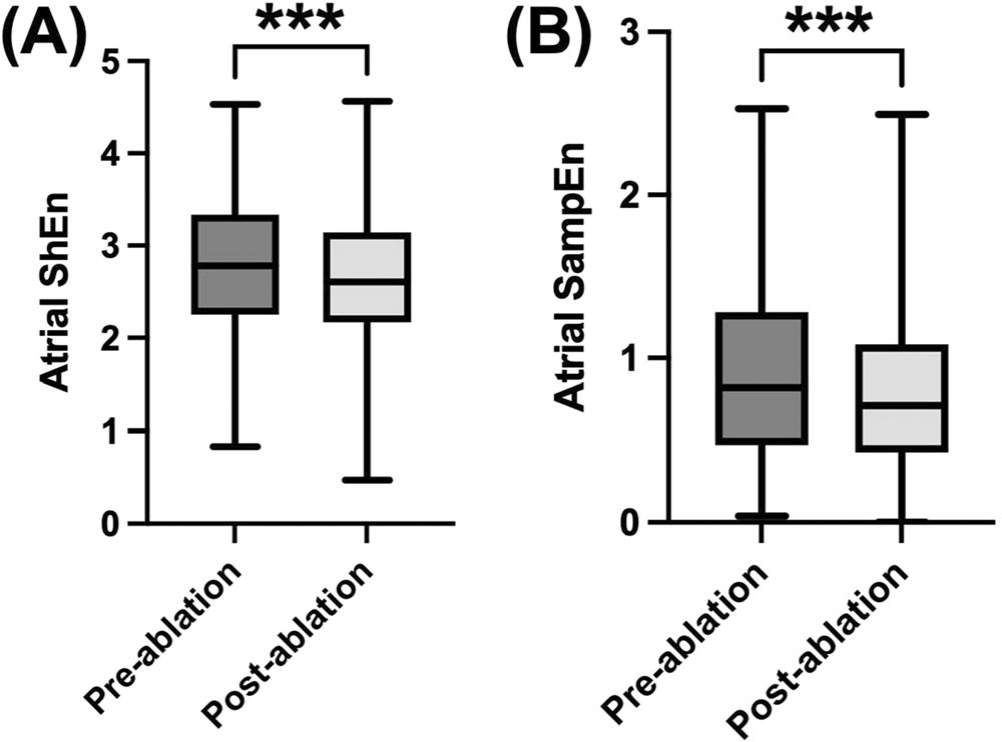
Impact of pulmonary vein isolation on atrial entropy. (A) Box and whisker plot of the median, interquartile range and range of coronary sinus channel (CS) 3‐4 Shannon entropy (ShEn) before and after ablation. (B) Box and whisker plot of the median, interquartile range, and range of CS 3‐4 Sample entropy (SampEn) before and after ablation. Differences between pre‐ and post‐ablation ShEn and SampEn were analyzed using a paired *t*‐test (*n* = 171). ***p < 0.001.

A significant decrease in atrial ShEn was observed following ablation across all CS channels, suggesting an increase in AF organization following ablation (CS 3‐4: pre‐ablation = 2.796 ± 0.707 vs. post‐ablation = 2.628 ± 0.685, *p* < 0.001, Figure 3A). Similar to ShEn, a significant decrease in atrial SampEn was observed following ablation across all CS channels (CS 3‐4: pre‐ablation = 0.907 ± 0.512 vs. post‐ablation = 0.790 ± 0.446, *p* < 0.001, Figure 3B). All pre‐ and post‐ablation ShEn and SampEn values are reported in Table 1.

**Table 1:** Impact of pulmonary vein isolation on atrial entropy.

Atrial entropy parameter	CS channel	Pre‐ablation	Post‐ablation	*p* value
ShEn	CS 1‐2	2.796 ± 0.842	2.638 ± 0.810	*p* < 0.001
	CS 3‐4	2.796 ± 0.707	2.628 ± 0.685	*p* < 0.001
	CS 5‐6	3.046 ± 0.700	2.867 ± 0.731	*p* < 0.001
	CS 7‐8	3.105 ± 0.698	2.877 ± 0.778	*p* < 0.001
	CS 9‐10	2.997 ± 0.724	2.739 ± 0.795	*p* < 0.001
SampEn	CS 1‐2	0.916 ± 0.591	0.814 ± 0.539	*p* < 0.001
	CS 3‐4	0.907 ± 0.512	0.790 ± 0.446	*p* < 0.001
	CS 5‐6	1.107 ± 0.541	0.980 ± 0.514	*p* < 0.001
	CS 7‐8	1.166 ± 0.523	1.014 ± 0.551	*p* < 0.001
	CS 9‐10	1.092 ± 0.549	0.910 ± 0.544	*p* < 0.001

*Note:* Atrial entropy values are shown as mean ± standard deviation.

Abbreviations: CS, coronary sinus; SampEn, Sample entropy; ShEn, Shannon entropy.

### 3.2 Correlation Between Atrial Entropy and RRI Variability and Irregularity

**Figure 4:**
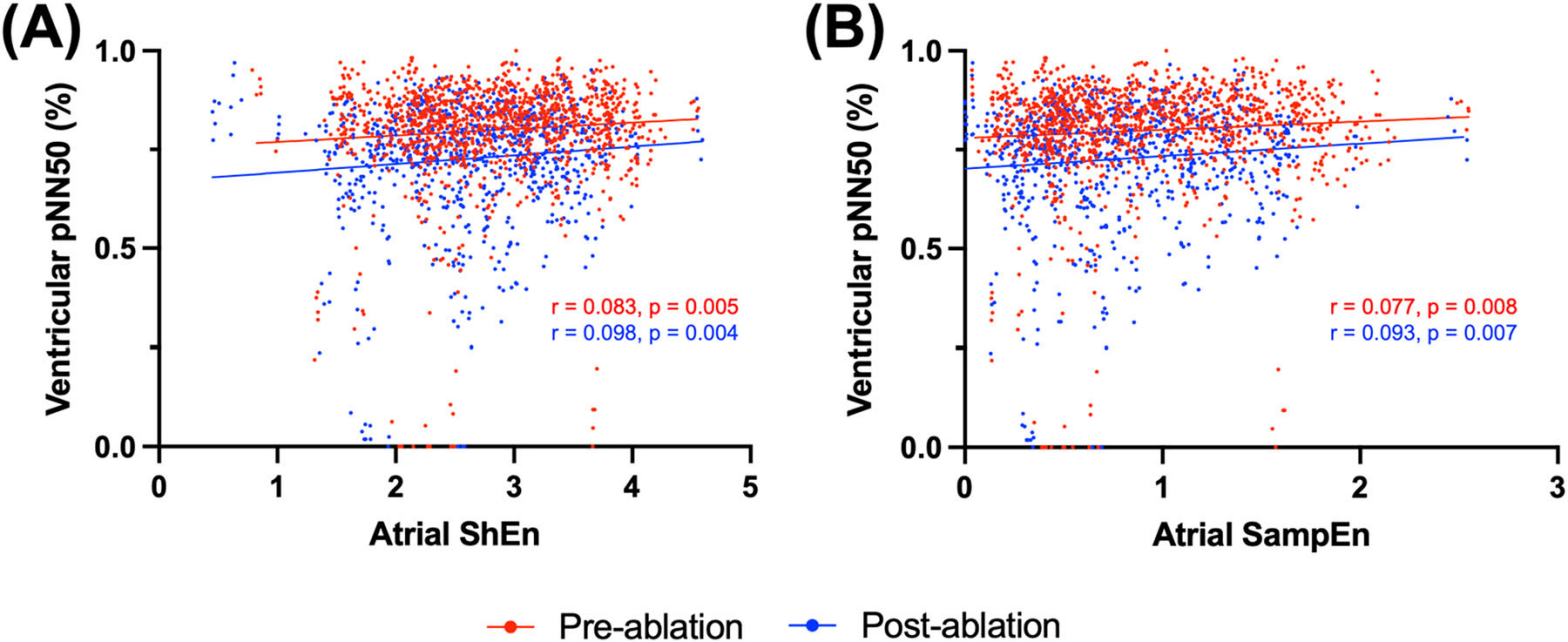
Correlation between atrial entropy and RR interval (RRI) variability as measured by pNN50. (A) Correlation between atrial coronary sinus channel (CS) 3‐4 Shannon entropy (ShEn) and ventricular pNN50. (B) Correlation between atrial CS 3‐4 Sample entropy (SampEn) and ventricular pNN50. Pearson correlation coefficients (*r*) given, and *p* values shown to indicate statistical significance of correlations (pre‐ablation *n* = 239, post‐ablation *n* = 171). All *p* values < 0.05 were considered statistically significant. pNN50 = percentage of successive RR intervals that differ from each other by more than 50 ms.

Atrial ShEn was significantly correlated with ventricular pNN50, normalized SD RRI and normalized mean RRI difference, suggesting the degree of AF organization was reflected in ventricular RRI variability (pNN50: pre‐ablation *r* = 0.083, *p* = 0.005 and post‐ablation *r* = 0.098, *p* = 0.004 (Figure 4A)*;* normalized SD RRI: pre‐ablation *r* = 0.174, *p* < 0.001 and post‐ablation *r* = 0.113, *p* < 0.001; normalized mean RRI difference: pre‐ablation *r* = 0.144, *p* < 0.001 and post‐ablation *r* = 0.124, *p* < 0.001; correlations with atrial ShEn CS 3‐4 shown). However, atrial ShEn was not found to be significantly correlated with ventricular rMSSD and SD RRI. All correlations between atrial ShEn and RRI variability measures can be found in Table 2.

**Table 2:** Correlations between atrial ShEn and measures of RRI variability and irregularity.

RRI parameter	CS channel	Pre‐ablation			Post‐ablation		
		Correlation coefficient (r)	*p* value	Significance	Correlation coefficient (r)	*p* value	Significance
Normalized mean RRI difference	CS 1‐2	0.098	< 0.001	***	0.100	0.003	**
	CS 3‐4	0.144	< 0.001	***	0.124	< 0.001	***
	CS 5‐6	0.117	< 0.001	***	0.144	< 0.001	***
	CS 7‐8	0.125	< 0.001	***	0.127	< 0.001	***
	CS 9‐10	0.124	< 0.001	***	0.127	< 0.001	***
SD RRI	CS 1‐2	0.055	0.056	ns	0.010	0.772	ns
	CS 3‐4	0.039	0.183	ns	0.027	0.433	ns
	CS 5‐6	0.007	0.817	ns	0.020	0.558	ns
	CS 7‐8	0.016	0.597	ns	0.008	0.811	ns
	CS 9‐10	−0.026	0.382	ns	−0.030	0.381	ns
Normalized SD RRI	CS 1‐2	0.124	< 0.001	***	0.100	0.003	**
	CS 3‐4	0.174	< 0.001	***	0.113	< 0.001	***
	CS 5‐6	0.126	< 0.001	***	0.152	< 0.001	***
	CS 7‐8	0.163	< 0.001	***	0.146	< 0.001	***
	CS 9‐10	0.138	< 0.001	***	0.127	< 0.001	***
rMSSD	CS 1‐2	0.048	0.099	ns	0.013	0.709	ns
	CS 3‐4	0.030	0.304	ns	0.032	0.351	ns
	CS 5‐6	−0.002	0.958	ns	0.020	0.568	ns
	CS 7‐8	0.014	0.641	ns	0.005	0.894	ns
	CS 9‐10	−0.031	0.295	ns	−0.032	0.350	ns
pNN50	CS 1‐2	0.121	< 0.001	***	0.104	0.002	**
	CS 3‐4	0.083	0.005	**	0.098	0.004	**
	CS 5‐6	0.078	0.007	**	0.141	< 0.001	***
	CS 7‐8	0.121	< 0.001	***	0.120	< 0.001	***
	CS 9‐10	0.117	< 0.001	***	0.107	0.002	**
SampEn	CS 1‐2	0.031	0.292	ns	0.134	< 0.001	***
	CS 3‐4	0.056	0.055	ns	0.123	< 0.001	***
	CS 5‐6	0.057	0.051	ns	0.129	< 0.001	***
	CS 7‐8	0.080	0.006	**	0.110	0.001	**
	CS 9‐10	0.049	0.093	ns	0.061	0.077	ns
Normalized SampEn	CS 1‐2	0.128	< 0.001	***	0.197	< 0.001	***
	CS 3‐4	0.166	< 0.001	***	0.180	< 0.001	***
	CS 5‐6	0.167	< 0.001	***	0.201	< 0.001	***
	CS 7‐8	0.196	< 0.001	***	0.210	< 0.001	***
	CS 9‐10	0.184	< 0.001	***	0.189	< 0.001	***

*Note:* Pearson correlation coefficient values given for correlations between atrial ShEn and the RRI variability parameters. Non‐parametric Spearman correlation coefficient values given for correlations between atrial ShEn and the RRI irregularity parameters: SampEn and normalized SampEn. *p < 0.05, **p < 0.01, ***p < 0.001, ns = not statistically significant.

Abbreviations: CS, coronary sinus; pNN50, percentage of successive RR intervals that differ from each other by more than 50 ms; rMSSD, root mean square of successive RR interval differences; RRI, RR interval; SampEn, Sample entropy; SD RRI, standard deviation of RR intervals; ShEn, Shannon entropy.

We observed significant correlations between atrial ShEn and ventricular normalized SampEn (CS 3‐4: pre‐ablation *r* = 0.166, *p* < 0.001 and post‐ablation *r* = 0.180, *p* < 0.001). Pre‐ablation, atrial ShEn and ventricular SampEn were significantly correlated in CS 7‐8 only, but no significant correlations between these two parameters were observed in the other CS channels. Post‐ablation, atrial ShEn was significantly correlated with ventricular SampEn in all channels except CS 9‐10. All correlations between atrial ShEn and RRI irregularity measures can be found in Table 2.

Similarly, atrial SampEn was significantly correlated with ventricular pNN50, normalized SD RRI and normalized mean RRI difference (pNN50: pre‐ablation *r* = 0.077, *p* = 0.008 and post‐ablation *r* = 0.093, *p* = 0.007 (Figure 4B)*;* normalized SD RRI: pre‐ablation *r* = 0.175, *p* < 0.001 and post‐ablation *r* = 0.108, *p* = 0.002; normalized mean RRI difference: pre‐ablation *r* = 0.144, *p* < 0.001 and post‐ablation *r* = 0.119, *p* < 0.001; correlations with atrial SampEn CS 3‐4 shown). However, atrial SampEn was not found to be significantly correlated with ventricular rMSSD and SD RRI. All correlations between atrial SampEn and RRI variability measures can be found in Table 3.

**Table 3:** Correlations between atrial SampEn and measures of RRI variability and irregularity.

RRI parameter	CS channel	Pre‐ablation			Post‐ablation		
		Correlation coefficient (*r*)	*p* value	Significance	Correlation coefficient (*r*)	*p* value	Significance
Normalized mean RRI difference	CS 1‐2	0.104	< 0.001	***	0.111	0.001	**
	CS 3‐4	0.144	< 0.001	***	0.119	< 0.001	***
	CS 5‐6	0.124	< 0.001	***	0.160	0.001	***
	CS 7‐8	0.143	< 0.001	***	0.138	< 0.001	***
	CS 9‐10	0.140	< 0.001	***	0.147	< 0.001	***
SD RRI	CS 1‐2	0.056	0.051	ns	0.028	0.420	ns
	CS 3‐4	0.047	0.106	ns	0.025	0.464	ns
	CS 5‐6	0.002	0.933	ns	0.027	0.431	ns
	CS 7‐8	0.014	0.629	ns	0.011	0.742	ns
	CS 9‐10	−0.024	0.418	ns	−0.018	0.593	ns
Normalized SD RRI	CS 1‐2	0.124	< 0.001	***	0.106	0.002	**
	CS 3‐4	0.175	< 0.001	***	0.108	0.002	**
	CS 5‐6	0.125	< 0.001	***	0.163	< 0.001	***
	CS 7‐8	0.177	< 0.001	***	0.147	< 0.001	***
	CS 9‐10	0.152	< 0.001	***	0.143	< 0.001	***
rMSSD	CS 1‐2	0.053	0.073	ns	0.032	0.344	ns
	CS 3‐4	0.036	0.216	ns	0.030	0.382	ns
	CS 5‐6	−0.001	0.784	ns	0.028	0.413	ns
	CS 7‐8	0.011	0.704	ns	0.011	0.750	ns
	CS 9‐10	−0.027	0.353	ns	−0.017	0.619	ns
pNN50	CS 1‐2	0.115	< 0.001	***	0.098	0.004	**
	CS 3‐4	0.077	0.008	**	0.093	0.007	**
	CS 5‐6	0.091	0.002	**	0.141	< 0.001	***
	CS 7‐8	0.133	< 0.001	***	0.127	< 0.001	***
	CS 9‐10	0.114	< 0.001	***	0.105	0.002	**
SampEn	CS 1‐2	0.031	0.283	ns	0.133	< 0.001	***
	CS 3‐4	0.061	0.036	*	0.122	< 0.001	***
	CS 5‐6	0.069	0.018	*	0.125	< 0.001	***
	CS 7‐8	0.084	0.004	**	0.104	0.002	**
	CS 9‐10	0.045	0.123	ns	0.067	0.050	ns
Normalized SampEn	CS 1‐2	0.124	< 0.001	***	0.195	< 0.001	***
	CS 3‐4	0.168	< 0.001	***	0.178	< 0.001	***
	CS 5‐6	0.173	< 0.001	***	0.209	< 0.001	***
	CS 7‐8	0.200	< 0.001	***	0.210	< 0.001	***
	CS 9‐10	0.182	< 0.001	***	0.204	< 0.001	***

*Note:* Pearson correlation coefficient values given for correlations between atrial SampEn and the RRI variability parameters. Non‐parametric Spearman correlation coefficient values given for correlations between atrial SampEn and the RRI irregularity parameters: SampEn and normalized SampEn. *p < 0.05, **p < 0.01, ***p < 0.001, ns = not statistically significant.

Abbreviations: CS, coronary sinus; pNN50, percentage of successive RR intervals that differ from each other by more than 50 ms; rMSSD, root mean square of successive RR interval differences; RRI, RR interval; SampEn, Sample entropy; SD RRI, standard deviation of RR intervals.

We observed significant correlations between atrial SampEn and ventricular normalized SampEn (CS 3‐4: pre‐ablation *r* = 0.0.168, *p* < 0.001 and post‐ablation *r* = 0.178, *p* < 0.001). Pre‐ablation, atrial SampEn and ventricular SampEn were significantly correlated in CS 3‐4, CS 5‐6, and CS 7‐8, but no significant correlations between these two parameters were observed in the remaining CS channels. Post‐ablation, atrial SampEn was significantly correlated with ventricular SampEn in all channels except CS 9‐10. All correlations between atrial SampEn and RRI irregularity measures can be found in Table 3. Overall, these findings were in line with the correlations observed between atrial ShEn and measures of RRI variability and irregularity.

## 4 Discussion

In patients undergoing catheter ablation for AF, we observed a significant increase in AF organization following PVI and found AF organization was significantly correlated with ventricular (RRI) variability and irregularity.

### 4.1 Increase in AF Organization Following PVI

Following PVI, both atrial ShEn and SampEn decreased supporting a mechanistic relationship between PVI and AF organization when measured after pulmonary vein exit block but before cardioversion. A possible explanation for the increased organization following PVI is that the reduction of the area of propagation in the atrium after isolation of the pulmonary veins leads to an improvement in spatiotemporal correlation of electrograms, leading to reductions in measurable entropy [28].

### 4.2 Mechanisms Regulating Ventricular Variability and Irregularity in AF

The significant correlations found in this study between atrial entropy and RRI metrics suggest that the degree of AF organization impacts the ventricular variability and irregularity on the surface ECG. The irregularity of the ventricular rhythm in AF is thought to be due to concealed conduction where rapid uncoordinated arrival of atrial impulses at the AV node leads to varying degrees of AV nodal penetration and refractoriness [29, 30]. In line with this mechanism, in organized electrophenotypes where AF may be driven by more stable sources of electrical activity, AV nodal penetration of the atrial impulses is likely to be more regular, therefore resulting in less ventricular variability and irregularity [29, 30]. Conversely, in disorganized AF, which may be sustained by multiple randomly propagating electrical wavelets, there is likely to be more varied AV nodal penetration resulting in more varied AV nodal refractoriness and conduction, and therefore higher ventricular variability and irregularity [29, 30]. The impact of PVI on the regularity of AV nodal penetration may explain the significant correlation between atrial entropy and RRI variability and irregularity, illustrating as AF becomes more organized ventricular variability and irregularity decreases. Interestingly, we found significant correlations between atrial entropy and ventricular normalized SD RRI and normalized SampEn both pre‐ and post‐ablation across all CS channels; however, this was not observed with non‐normalized ventricular SD RRI and SampEn. Normalization was performed to account for the impact of heart rate on RRI variability [25]. We hypothesize that the varying heart rates of the study participants may have masked any potential correlations between atrial entropy and ventricular SD RRI and SampEn. However, the magnitude of the correlations suggests AF organization is unlikely to be the only factor impacting ventricular variability and irregularity in AF. Other factors such as autonomic nervous system activity are likely to play a role, as vagal blockade was shown to reduce RRI variability in AF, however the extent of its impact was limited and a substantial degree of RRI variability remained [31]. Together with our results, this suggests that the AV node intrinsically produces an irregular ventricular rhythm in AF, the irregularity of which may be largely independent from external factors.

### 4.3 Clinical Implications

The discovery that atrial entropy is correlated with RRI variability and irregularity indicates that we have a non‐invasive means of assessing AF organization from surface ECG, that is likely more reliable than F‐wave assessment which is likely less accurate due to the influence of ventricular activity and noise [32]. This non‐invasive assessment may offer a quick, easy, and patient‐friendly approach to evaluating AF organization. Determining the level of AF organization before procedures might allow ablation strategies for AF to be better tailored to the electrophenotype present, which may in turn increase the success of the ablation procedure [2]. However, due to the magnitude of the correlations, RRI variability and irregularity on their own may not be sufficiently reliable indicators of AF organization. Further research is required to investigate if correlations exist between clinical outcomes downstream of PVI and AF organization phenotype.

### 4.4 Limitations

Firstly, the CS catheter only records local left atrial signals, therefore the CS electrograms are not necessarily representative of bi‐atrial activity [33]. Secondly, general anesthetic may have impacted the RRI variability measurements [34]. Thirdly, due to differences in anatomy and procedural factors, there may have been differences in CS catheter positioning between patients. Whilst this may have impacted the morphology of the CS electrograms and hence atrial entropy values, it also reflects the real‐life setting of this study [33]. Fourth, there is no gold standard for measuring AF organization that has been meaningfully linked to ablation strategies and outcomes to then be correlated with, with entropy being the best available. Finally, another limitation is the absence of re‐mapping after PVI. Based on the procedure reports, the AF did not organize into an atrial flutter/macro‐reentrant left atrial tachycardia following PVI, but we could not fully exclude this possibility in the absence of re‐mapping after PVI.

## 5 Conclusion

In this study, we found a reduction in atrial entropy following PVI, suggesting PVI is accompanied by a shift toward the organized spectrum in AF electrophenotype. Furthermore, we found that atrial entropy was significantly correlated with RRI variability and irregularity, suggesting that the degree of AF organization impacts ventricular response, which can be measured non‐invasively via the surface ECG. Further research to investigate whether non‐invasive interpretation of AF organization from RRI variability and irregularity may be associated with clinically meaningful outcomes is required.

## Ethics Statement

This retrospective cohort study was approved by the Regional Ethics Board (IRAS ID: 258686 and 293374).

## Conflicts of Interest

The authors declare no conflicts of interest.

## Supporting information

Supplement PVI AF Organisation.

## Data Availability

The data that support the findings of this study are not publicly available owing to ethical restrictions.
