# Supplementary material for: Impact of Pulmonary Vein Isolation on Atrial Fibrillation Organisation: Correlation of Intracardiac and Surface Electrocardiogram Measures
Source: J Cardiovasc Electrophysiol. 2025 Aug 6;36(10):2529–38. doi: 10.1111/jce.70044 (PMC12530675; doi:10.1111/jce.70044)
Supplement: Supplementary file 1 — Supplement PVI AF Organisation. [file JCE-36-2529-s001.docx]

**Supplement**

**Description of RR interval variability time-domain measures**

Noramlised mean RRI interval (RRI) difference, measured in milliseconds (ms), was obtained by first calculating the time differences between successive RR intervals. Each time difference value is then divided by the value of its previous RR interval and the subsequent results are averaged. This is summarised using the following equation: *Normalised mean RRI difference = Mean (RRI difference/preceding RRI)*.

Standard deviation of RRIs (SD RRI) is measured in ms.

Root mean square of successive RR interval differences (rMSSD), measured in ms, was obtained by first calculating each successive time difference between heartbeats. Then each of the values is squared and the mean of these is calculated. Finally, a square root of the total is obtained. This is summarised using the following equation: *rMSSD =√[Mean (RRI difference)^2^].*

pNN50 is the percentage of successive RRIs that differ from each other by more than 50ms.
